# Investigating antiquities trafficking with generative pre-trained transformer (GPT)-3 enabled knowledge graphs: A case study

**DOI:** 10.12688/openreseurope.16003.1

**Published:** 2023-06-20

**Authors:** Shawn Graham, Donna Yates, Ahmed El-Roby

**Affiliations:** 1 Department of History, Carleton University, Ottawa, Ontario, Canada; 2 Faculty of Law, Universiteit Maastricht, Maastricht, Limburg, 6211LH, The Netherlands; 3 School of Computer Science, Carleton University, Ottawa, Ontario, Canada

**Keywords:** Large language models, gpt3, knowledge graph, knowledge graph embedding model, antiquities trade, illicit antiquities, art market, antiquities trafficking

## Abstract

**Background:** There is a wide variety of potential sources from which insight into the antiquities trade could be culled, from newspaper articles to auction catalogues, to court dockets, to personal archives, if it could all be systematically examined. We explore the use of a large language model, GPT-3, to semi-automate the creation of a knowledge graph of a body of scholarship concerning the antiquities trade.

**Methods:** We give GPT-3 a prompt guiding it to identify knowledge statements around the trade. Given GPT-3’s understanding of the statistical properties of language, our prompt teaches GPT-3 to append text to each article we feed it where the appended text summarizes the knowledge in the article. The summary is in the form of a list of subject, predicate, and object relationships, representing a knowledge graph. Previously we created such lists by manually annotating the source articles. We compare the result of this automatic process with a knowledge graph created from the same sources via hand. When such knowledge graphs are projected into a multi-dimensional embedding model using a neural network (via the Ampligraph open-source Python library), the relative positioning of entities implies the probability of a connection; the direction of the positioning implies the *kind* of connection. Thus, we can interrogate the embedding model to discover new probable relationships. The results can generate new insight about the antiquity trade, suggesting possible avenues of research.

**Results:** We find that our semi-automatic approach to generating the knowledge graph in the first place produces comparable results to our hand-made version, but at an enormous savings of time and a possible expansion of the amount of materials we can consider.

**Conclusions:** These results have implications for working with other kinds of archaeological knowledge in grey literature, reports, articles, and other venues via computational means.

## Plain language summary

There is a wide variety of potential materials one could study to shed light onto the antiquities trade; the problem is both to read and systematically make sense of that mass of material. The article discusses the use of a large language model called GPT-3 to semi-automate the creation of a systematic representation of the trade as a ‘knowledge graph’. GPT-3 summarizes specialist articles about the antiquities trade through the creation of statements consisting of subject, verb, object triplets. These statements can be knit together as a network or ‘knowledge graph’. The authors compare the results of the semi-automatic process with a hand-made knowledge graph created from the same sources. By projecting the knowledge graph through a neural network to create a multi-dimensional embedding model (where different kinds of ‘knowledge’ captured by the graph represents different directions in the model’s space), the authors can examine the relative positioning of entities such that the closer entities are in this embedding model, the greater the likelihood of a real-world connection between them. This allows the authors to generate new ‘leads’ to investigate in the antiquities trade, from patterns in the existing knowledge otherwise invisible. The authors find that their semi-automatic approach produces comparable results to the hand-made version but with a significant savings of time and a potential for considering more materials. The authors suggest that their findings could have implications for other archaeological knowledge in various venues via computational means.

## Introduction

The systematic retrieval of knowledge about any field is hampered by the wide variety of ‘containers’ for that knowledge. In the context of archaeology, these containers can be field reports, field diaries, formal papers, monographs, blog posts, video transcripts, and grey literature of all kinds, some of which is digitized, much of which is not. Accessing this information systematically continues to be a problem for archaeology. In 2015, Kintigh pointed out how all of archaeology’s so-called ‘grand challenges’ that he and his coauthors identified ( Kintigh *et al.,* 2014) rest on the ability to extract knowledge about a domain, no matter where that knowledge might reside, and he could offer no examples of automatic extraction of archaeological domain knowledge at that time ( Kintigh, 2015). Since then, with the development of language models of various kinds, this state of affairs is rapidly changing.

The invention of technologies such as ‘BERT’ by Devlin *et al.* (2019) was one of the first inklings of this coming change. ‘BERT’ stands for Bidirectional Encoder Representations from Transformers, which uses a particular neural network architecture to learn the grammatical structure of text for a particular domain from manually annotated training data. ^1^ In archaeology, Brandsen *et al.* (2021) developed a version of a BERT-type model on a corpus of Dutch archaeological reports so that the model learned to identify concepts in Dutch archaeology. They trained their model on over 60,000 reports drawing on 15 manually annotated articles containing 43,000 annotated entities ( Brandsen *et al.,* 2021: 6) and found that the resulting model worked well. This experience points to one of the limitations of this kind of specially-trained model approach: the need for extensive well-formatted training data.

In our case, we wanted to achieve something similar in the domain of antiquities trafficking for an experiment presented in Graham *et al.* (2023) and discussed below, but we had nowhere near the required amount of training data to take this approach. Indeed, many potential archaeological applications with these special-purpose models will founder on the rocks of not enough training data. At around the time that the article outlining the results of that experiment entered submission, the emergence of ‘generative pre-trained transformer’ (GPT) architectures in 2018 ( OpenAI, 2018; Radford *et al.,* 2018) offered a different way forward. These GP’ models (also called ‘large language models’) have impressive capacities to ‘learn’ how to do tasks for which they have not been explicitly trained, as a function of both their architecture and the enormous volumes of texts they were initially trained on. The so-called ‘zero-shot learning’ capability caught our attention because it suggests that we do not need to develop a training corpus (see below). We had just conducted an experiment which required manually annotating over 100 articles with subject, object, and predicate relationships taking several months of work, but only identifying a few hundred entities (see Graham *et al.,* 2023). With GPT, we saw that there was great potential to automate the annotation of useful texts in this domain saving months of work. We also hoped that using GPT would identify more relationships and entities in our texts than we caught by eye, and catch them more consistently, thus improving the overall quality of our final output, a knowledge graph embedding model.

In this paper, we detail an experiment using a particular large language model (GPT-3 from OpenAI, 2020) to automatically extract information into semi-structured form. For our experiment we are using the same series of online articles concerning the antiquities trade as described and published by the Trafficking Culture Project that we used in our earlier, manually supported experiment. ^2^ The extracted information is represented as a series of statements or ‘triples’ in subject, verb, object format. Such statements can be knit together to make a network or knowledge graph of facts as recounted in those articles. That knowledge graph can then be transformed so that we can interrogate it for likely relationships (the knowledge graph embedding model). Our purpose in this paper is to evaluate the results of this use of GPT-3 for automated ‘zero-shot’ extraction.

Large language models (LLMs), like the one we have employed here, are often colloquially referred to as ‘artificial intelligence’. To be clear, a large language model does not know anything; it is a statistical representation across billions of parameters of patterns of word use in texts scraped from the internet ( Bender *et al.,* 2021), where the words are represented as ‘tokens’ that encode basic units of text or characters which get assigned numerical values. ^3^ In a way, LLMs are extraordinarily powerful auto-complete software; they create text that looks like what one might expect for a given situation. For a zero-shot approach, one prompts the model to produce the kind of text one wants to see; for instance, one could instruct the LLM to ‘Extract relationships from the following text as subject, predicate, object triples’ (our actual prompt is somewhat more complicated, see below). A slightly more effective approach is to devise a prompt that combines a clear instruction with an example of the desired output or, rather, the desired auto-completion. This is called ‘one shot learning’ in that we only give the model one example of what we are after. ^4^ This prompt guides the model to the statistical space within the large language model where if one were to read X, the appropriate response would look like Y, as displayed by our prompt.

To test the use of a large language model as an alternative to manually building a workable knowledge graph about the antiquities trade, we experimented with the ‘sandbox’ environment from OpenAI and examined blogposts and other literature where people share prompting advice. We devised a prompt that, when tested on short samples of text from the Trafficking Culture website, seemed to capture what we were after. A more systematic approach to devising such a prompt might be to generate a large range of candidate prompts, and then to iterate through these on the same small body of text to identify the landscape of possible results such that a best-case prompt could be; this strikes us as similar to how researchers using agent-based models ‘sweep’ through different parameters to understand the ‘behaviour space’ of their models (see for instance Romanowska *et al.,* 2021: 313–325). Further research is needed in terms of so-called ‘prompt engineering’ to find the most effective ways of leveraging that power.

All told, we evaluate the success of the experiment by completing the entire analytical process from our earlier paper using the automatically generated knowledge graph. We transform the knowledge statements into a knowledge graph embedding model which uses a neural network to turn statements into mathematical vectors which can be measured for similarities. We compare the descriptive statistics for the knowledge graph embedding model derived from this automated knowledge graph with the model and its predictions from our earlier experiment that we built manually ( Graham *et al.,* 2023) for speed, accuracy, and usability. We believe that this has important implications for the use of our previously-proposed methods for researchers and investigators. This is something that can be realistically employed in other domains as well.

In the following sections, we recount the experiment and detail our process so that others might employ a similar approach using GPT-3 or its successor/competitor models. The pace of development in the LLM space is rapid, and their deployment in multiple domains is happening faster than we can evaluate results; the present experiment’s codebase might well be rendered obsolete quite quickly. However, we believe that this experiment will help us understand the strengths and limitations of using large language models towards approaching social and archaeological questions moving forward; at the very least, the potential for liberating archaeological knowledge in computationally tractable ways seems promising.

## Our previous experiment: the baseline

The contours and facts about the illegal or illicit antiquities trade and its intersections with the licit trade emerge through a wide variety of media. This data comes from both a bewildering array of sources and in a myriad of formats. These include but are not limited to documents generated by the criminal justice system and courts (e.g., Bogdanos, 2021; District Attorney New York County, 2019), media reports and investigations (e.g., Felch & Frammolino, 2011; Sabar, 2020; Watson & Todeschini, 2007), independent blog-published investigations (e.g., see Gopinathan, 2021; Jayaraman, 2015; Krishnankutty, 2022)) ethnographic research (e.g., Kersel, 2006a; Kersel, 2006b; Paredes Maury, 1999), exhibition catalogues (e.g., Chippendale & Gill, 2000), auction data (e.g., Beltrametti & Marrone, 2016; Brodie, 2019; Davis, 2011; Fabiani & Marrone, 2021; Gilgan, 2001; Levine & Martínez de Luna, 2013; Yates, 2006), and provenance and police databases (e.g., Oosterman, 2018; Oosterman, 2019). None of these sources of information have been developed with machine readability or interoperability in mind.

In our previous experiment, we organised a body of short articles about the antiquities trade ^5^ into a series of simple statements of the kind ‘medici, sold to, hecht’ ^6^, annotating each article by hand and thus drawing connections between subjects and objects by determining the relationship. The result was a network graph capturing our distilled vision of the body of knowledge in those articles. Network analysis methods can provide insight about cliques and subgroups and give an idea of a person, organization, or object’s relative importance through those connections. But network analysis does not, and for the most part cannot, say anything about the missing links or the gaps in that network—the connections that do not directly exist within the data available. However, a technique called ‘knowledge graph embedding models’ can give us a basis for making suggestions about the possible real world links between people and other entities in a network which are not explicitly stated in the source data. This enables us to predict connections that are likely to exist, given not just the pattern of connections, but also the semantic meanings of those connections. Those suggested connections can then be followed up on via traditional investigative research. This was the technique we employed in our first exploration ( Graham *et al.,* 2023).

In this technique, the statements in the network are projected through a neural network model to become vectors in a multidimensional space; the description of how each statement perturbs the network is represented mathematically as a list of numbers or a vector describing the direction that statement takes through the multidimensional space described by the neural network (the ‘embedding space’). The distance between vectors can be measured and that distance can be used to estimate the likelihood of a hypothesized link. In our previous experiment, we asked the computer to take all of the actors listed in our network to create hypotheses around various predicates like ‘sold to’, ‘partnered’, ‘bought from’. The list of hypotheses was then measured, and the most likely hypotheses were reported. One such hypothesis it suggested was a connection between the convicted illicit antiquities dealer Leonardo Patterson (see, for example, Mashberg, 2015) and the Brooklyn Museum, a possible connection hitherto unknown to us. DY examined the catalogue of the museum’s holdings and found two low-value Mexican antiquities donated to the museum by Leonardo Patterson. This prompted a larger investigation into Patterson’s relationship with museums during the 1960s through the 1980s that led to a trail of other low-value donations by Patterson and a pattern of possible ‘reputation laundering’ activities that had so far slipped under the radar. The intriguing research line that that resulted from our first foray into following up on a hypothesized link indicates that our approach may be a valuable one and adds a new tool to the investigator’s workbench.

Yet despite the apparent utility of the approach, getting to the point where we could generate hypotheses was time consuming and labor intensive. Converting the unstructured text (i.e., human-readable text, rather than text organised for machine-reading) into an annotated format suitable for computation took several months of work. Each article had to be annotated by hand, indicating the individuals, organizations, places, and objects and their interrelationships. Automating this type of work is an active area of research in the field of ‘Natural Language Processing’, a suite of techniques called ‘Named Entity Recognition’ (NER) and ‘Relation Extraction’. In a conventional automated approach, one might use an existing dictionary (or construct one from scratch) of named entities that would then be used to parse the unstructured text. However, if one does not know all of the relevant entities beforehand, or if there is any ambiguity in the text that one is analyzing, this approach might miss or improperly categorize important data. Other approaches might use pattern matching, or probabilistic completions given descriptions of the grammatical parts of words and so on. In our previous experiment, we found that these NER approaches were more difficult to implement than manual annotation and were very error-prone for our particular domain. It was easier to simply invest the time in doing the hand annotation, since our corpus was comparatively small (129 articles, each of a few thousand words at most).

Realistically, then, this approach cannot scale and our hopes of developing our approach into a working tool for investigators in this field are contingent on finding a workable alternative to manual annotation. This is where the prospect of large language models appears useful. The statistical description of the knowledge graph embedding model created from our hand-annotated corpus of materials is the baseline against which we evaluate the experiment presented in the subsequent sections.

## Large language models

The emergence of so-called large language models (LLM) might offer us a short-cut and allow us to invest our time and energy elsewhere in the analytical process, sparing us the need for time consuming manual annotation in advance of projecting a knowledge graph. As indicated earlier, fundamentally LLMs are prediction engines; given a pattern of letters, they answer the question “what are the most likely next letters?” What gives LLMs their power is the sheer mass of text that they have been trained on. Once a training dataset passes a certain size in a neural network using a GPT architecture, it seems that there are emergent effects that transform what the model can accomplish. If we ask an LLM to predict the next sequence of letters, but also give it a pattern for what that predicted sequence should look like (that is, we employ a one-shot learning approach), the LLM is able to do what it has not been explicitly designed to do: it has learned to generalize from general patterns of word sequence to generate words that follow a general pattern.

The following is an example of a so-called ‘one shot’ prompt using OpenAI’s sandbox interface to access GPT-3:

        <prompt>

        > Create a table listing entities and relationships in a text.

        >

        > Text: In 2022 Donna Yates knocked over the vase she received as a gift from a friend.

        >

        > |object|date|individual|action|

        > |-------|-----|-----------|-------|

        > |vase|2022|Yates|broke|

        >

        > Text: Medici started dealing in antiquities in Rome during the 1960s. In July 1967, he was convicted in Italy of receiving looted artefacts, though in the same year he met and became an important supplier of antiquities to US dealer Robert Hecht. In 1968, Medici opened the gallery Antiquaria Romana in Rome and began to explore business opportunities in Switzerland. It is widely believed that in December 1971 he bought the illegally-excavated Euphronios (Sarpedon) krater from tombaroli before transporting it to Switzerland and selling it to Hecht. ^7^

Our prompt is a combination of a description of the output we want, an example text and the formatted correct result, and then the next piece of text to process. GPT-3 will read that text (‘…selling it to Hecht.’) and understand that the next tokens to create will be those to represent a table:

        <result>

        > |Object|Date|Individual|Action|

        > |------|----|----------|-------|

        > |Antiquities|1960s|Giacomo Medici|Started dealing in|

        > |Antiquities|July 1967|Giacomo Medici|Convicted of receiving looted artefacts|

        > |Antiquities|1968|Giacomo Medici|Opened Antiquaria Romana in Rome|

        > |Euphronios (Sarpedon) Krater|December 1971|Giacomo Medici|Bought illegally-excavated|

        > |Euphronios (Sarpedon) Krater|December 1971|Giacomo Medici|Transported to Switzerland|

        > |Euphronios (Sarpedon) Krater|December 1971|Giacomo Medici|Sold to Robert Hecht|

In this example, we told GPT-3 what we wanted it to do, we gave it an example text, and we showed it how to organise the results. In its generated text, GPT-3 correctly formatted the results using the markdown table format and pulled the source text apart correctly.

While it may seem as if GPT-3 knows what it is doing, in truth it does not ‘know’ anything; an LLM is a token sequence predictor (GPT and similar models do not represent text directly, but rather encodings or compressions of text called ‘tokens’). Murray Shanahan, approaching LLMs from a philosophical perspective, makes the issues clear:

“It could perhaps be argued that an LLM “knows” what words typically follow what other words, in a sense that does not rely on the intentional stance. But even if we allow this, knowing that the word “Burundi” is likely to succeed the words “The country to the south of Rwanda is” is not the same as knowing that Burundi is to the south of Rwanda. To confuse those two things is to make a profound category mistake. If you doubt this, consider whether knowing that the word “little” is likely to follow the words “Twinkle, twinkle” is the same as knowing that twinkle twinkle little. The idea doesn’t even make sense.” ( Shanahan, 2022)

LLMs have famously been called ‘stochastic parrots’ ( Bender *et al.,* 2021) meaning they have no access to meaning. LLMs have no external access to the world, and so cannot form beliefs about the world. However, other work by Li *et al.* (2022), through experimental probing of a LLM trained on sequences of moves in the Othello board game, finds indications of some kind of ‘emergent nonlinear internal representation of the board state’. In other words, the model does have some kind of sense (although maybe not something sensible to a human) of the state of the world from which it draws its predictions. LLMs are black boxes whose workings remain impenetrable to us, but as they scale larger, each iteration seems capable of more varied and more complex tasks.

Normally, with a neural network, getting it to perform a new task would involve retraining the network on a vast amount of new data. Some research suggests that given the scale of data that GPT3 and similar models have been trained on in the first place, there are linear regression models already present inside the larger model ( Akyürek *et al.,* 2022). What might be happening therefore is that our example text prompts the model to find the linear regression model inside it that is closest to our example, and then to slightly retrain that internal model for the task at hand. This gives us grounds for believing that when we devise this knowledge extraction task for the model using a one-shot approach, GPT-3 is producing meaningful results, not just results that ‘look like’ what we might expect.

Work exploring and evaluating GPT-3’s general model of language against other fine-tuned models or tools for various natural language evaluation tasks like classification, paraphrasing, or data annotation suggested to us that GPT-3 would be broadly successful, if not as accurate, as manual annotation. The potential speed of the LLM might be worth the trade-off of decreased accuracy. Huang *et al.* (2023) categorized hate speech automatically with GPT, also asking the model to provide explanations or rationales for why it classified a particular social media post as hateful. They found that while the LLM did not identify as many instances of hate speech as the human annotators (identifying 80% of the test data correctly as hateful), they found its explanations for why a tweet might be hateful to be better than those provided by the humans. Kuzman *et al.* (2023) used GPT-3 to identify the genre of short texts, comparing the general GPT-3 model to a more specialized fine-tuned model created from manually annotated texts explicitly identifying genre. In their experiment, they found the generalist GPT-3 model performed better than the specialist model and was more effective at the task, when both were used on a dataset that neither GPT-3 nor the fine-tuned model had ever seen before. Gilardi *et al.* (2023) set out to evaluate the quality of GPT-3 explicitly for annotation for natural language processing tasks, comparing its results with those obtained from human workers (via Amazon’s Mechanical Turk service) and found that GPT-3 generally performed better. ^8^ Given these results, we felt reasonably confident that in our use case the results would be fairly comparable if not better than what we had achieved by hand.

So, large language models are prediction engines for the next likely token given a particular input, and they seem to contain within themselves some kind of representation of the/a world. LLMs might be better understood, from a humanistic perspective, as competing models of culture ( Underwood, 2022), and we can retrain such models on the different aspects of culture that we might wish to explore. In which case, instead of using Named Entity Recognition, which, as discussed above, depends on us knowing possible named entities in advance, we could tell GPT-3 that we want it to extract the names, places, organizations, and objects in the unstructured text we give it, and we want it to write this information out for us in such a way that we can then create a knowledge graph embedding model to predict new hypotheses about the antiquities trade.

## Method

To test the feasibility of using a large language model to create the initial knowledge graph for subsequent projection into a knowledge graph embedding model, we devised the following experiment. Using the same corpus of materials as our initial manually generated model, namely the 129 articles from the Trafficking Culture Encyclopedia ( https://traffickingculture.org/encyclopedia/all/), we present each one in turn to a large language model that has been prompted to identify subject, predicate, and object relationship triples for a given sentence. We then compile those results and project that graph through a neural network (creating the embedding model) and measure its properties and some of its hypotheses.

Essentially, our experiment involves four steps:

1. Create a suitable prompt.
2. Extract structured statements about the antiquities trade from unstructured text.
3. Create the knowledge graph embedding model
4. Test for new hypotheses.

In our first exploration, we performed step two by hand using some specialized tools to ensure consistency in the annotations that we were performing. In a sense, step *one* happened when we instructed our graduate students in what we wanted them to do, showing them how to annotate a text, showing them how the annotation platform worked, configuring and setting up individual access, and reconciling each student’s approach so that the resulting annotations would be consistent. This took several months’ worth of our students’ available time.

### Preprocessing the text

Perhaps this should be ‘step 0’. Preprocessing is the necessary underpinning of all computational work on text; it is the reformatting of text so that it can be computationally analyzed. The articles we are using are available in the ‘Encyclopedia’ on the Trafficking Culture website. We scraped those articles, removing html and in-text citations and reference lists. GPT-3’s application programming interface (API) has a limit on the number of tokens that it can process at any one time. ^9^ A token represents approximately four characters in a file. Large texts that that exceed the token limit will not be processed. Thus, we cannot simply feed each article one at a time to GPT-3 for processing, as larger files would exceed the limits. ^10^

Instead, after some experimentation we found that our prompt and around six kb’s worth of text would be the maximum amount of text we could pass to GPT-3 each time without fear of error. We wrote a small program to solve the problem (available in our code repository). The program examines each file in a folder, checking to make sure there is no file over six kb; it splits larger files until the results are under six kb in size. Obviously, this introduces one possible source of error into our larger experiment: relationships that span the break in the file that we introduce. ^11^

### Creating a suitable prompt

In the first few months of 2023 (the time of writing), a veritable cottage industry of ‘how to prompt’ guides has emerged; a quick web search will return hundreds of examples. Because there is no overarching theory of how LLMs work in the sense that one can systematically link a given prompt with a particular kind of output, prompt development feels rather like performing alchemy. Weng (2023) writes,

“Many studies looked into how to construct in-context examples to maximize the performance and observed that **choice of prompt format, training examples, and the order of the examples can lead to dramatically different performance**, from near random guess to near SoTA [state-of-the-art].” (Emphasis in the original).

That said, certain techniques have emerged that seem to provide some consistency in terms of high quality outputs. So-called ‘few-shot’ prompts can be extremely effective because they give the model a few examples of the desired possibility space. The examples should match closely both the input text and the desired result. A related approach, ‘instruction prompting’ simply summarizes the implied instructions that showing several examples would convey. The first approach takes more tokens to implement meaning less text can be processed by the API in a given call, while the second approach allows the model latitude in creation of the results. For our experiment we elected to combine both approaches so that we would get the flexibility of an instruction (and presumably, more relationships captured) with output that would not require much subsequent transformation to be useable.

We adapt a program written by Sixing Huang (2022) to pass our prompt and the target text one at a time to GPT-3 (our adaption involves changing the prompt and modifying it to gently retry if there are any errors so that we do not abuse the OpenAI service). This enables us to observe results as they are generated such that we can error check more easily. We have GPT-3 write the results in json notation, where there are consistent keys and values. This enables us to store the data in a graph database like Neo4j. ^12^ We also took inspiration from the Promptify python package (promptify.readthedocs.io) and Varun Shenoy’s ‘GraphGPT’ tool ^13^ for the creation of our prompt, examining the prompts they used in terms of how they framed their instructions to find a productive framing; this involved some experimentation. ^14^

Our eventual most successful prompt:

        ```

        You are given unstructured text about the antiquities trade. Extrapolate as many relationships as you can from the prompt concerning individuals, organizations, places, and objects. Every node has a name, label. Every edge has a to and from with node names, and a label. Edges are directed, so the order of the from and to is important. Format as json.

        Example: Mary Turlington made a ceramic Ducky in Big Pond in 2023; she had Kenny Wong paint it. It was bought by the Grand Narrows Museum in 2023 for $123456.

        [{

               "nodes": [{

                           "label": "the Ducky",

                           "type": "Object",

                           "madeIn": "Big Pond",

                           "date": "2023",

                           "potter": "Mary Turlington",

                           "painter": "Kenny Wong",

                           "price": "$123456"

               }],

               "edges": [{

                           "startNode": "the Ducky",

                           "endNode": "Grand Narrows Museum",

                           "type": "bought by",

                           "date": "2024"

               }]

        }]

        ```

Note in our prompt we do not describe *all* of the entities; it is sufficient to describe just one. Note also the use of the definite article ‘the’ in the label for the one entity. We have found that without using definite articles, GPT-3 can get confused and reverse the direction of action. Because GPT-3 will sometimes return the prompt text in certain conditions it is important that our prompt contains people, objects, locations, and so on that we know are NOT present and cannot be present in, in our case, the antiquities trade. Experimentation suggests that this happens when GPT-3 has no suitable completion available but it has not yet returned the (user-set) desired number of tokens, thus it must return something.

In our example, we have used characters and places from the Vinyl Cafe stories by Canadian raconteur Stuart McLean. When we get the output from GPT3, if we find any instances of ‘Mary Turlington’ we know that here was a moment when GPT-3 could not find anything in the text to extract but was still compelled to generate something because of the number of characters of text we requested be returned. The ‘temperature’ or creative latitude we give GPT-3 is set to 0. By setting such a low temperature, GPT-3 is strongly constrained to just our target text and not its larger training data set.

Our code for passing the unstructured text and prompt is adapted from that written by Huang (2022) and may be found in the accompanying repository. It requires a Python 3.9 environment with the OpenAI API Python wrapper installed, and a ‘key’ from an account tied to a credit card with OpenAI. We used the proprietary GPT-3 model because its API, behaviour, and results are currently the best developed and understood. However, open source LLMs are now being developed, and so the general approach we use here should translate to those LLMs in due course.

### Cleaning

The resulting completions are written to an output file. We remove duplicates, check for our example prompt text, and make sure that when individuals are named that both forename and surname are present and that if acronyms are used for organizations like the FBI, we use them consistently (either FBI or Federal Bureau of Investigation, but never both). Pattern searches in a text editor can accomplish this quite quickly.

### Graph database

The output from the model is organized in key:value pairs. We wrap this in the conventions of a .json file. This permits us to import the results into a graph database engine like Neo4j for future research questions.

### Knowledge graph

We take the list of relationships or edges and organize them as a simple csv file with three columns: start node (entity), relationship type, and end node. Again, this can be accomplished in a matter of seconds using a text editor and a simple search and replace. This file is now ready to be transformed into a knowledge graph embedding model via the Ampligraph Python package.

## Results

In our first exploration, annotating the articles and transforming the results into a csv table thence embedding model took us about three months to accomplish. With GPT-3, once we devised the prompt, the entire process took about three hours. The total cost was on the order of $50 (which included testing the prompt).

### Hallucinations and errors

We set the temperature variable to 0.1 to give the model a slim chance at a restricted ‘creativity’ sufficient to work out co-reference, that is, to identify which pronoun goes with what noun; when encountering a pronoun, we imagined that this level of creativity would help it identify the correct proper noun. There is as of yet no theoretical framework for understanding and working with large language models such that we can confidently predict what the outcome of particular prompts and parameters will achieve, making all such explorations rather more alchemical than scientific. Setting the temperature at 0.1 did leave us with a handful of instances where our cast of characters from our prompt made an appearance; Mary Turlington is an occasional art thief, it would seem. We deleted these statements.

There were some instances where the directionality of the relationship was misrecorded; that is, organization_a, sold_to, person_b should have read ‘person_b, sold_to, organization_a’ *or* ‘organization_a, bought_from, person_b’. There did not seem to be many of these, less than 5 percent of the total number of statements. Finally, there were instances where an individual might be named with their full name, and in other places, by their surname. Similarly, sometimes a collection would be named as ‘the Fleischman Collection’ and other times, ‘the Barbara and Lawrence Fleischman Collection’. We located such instances by sorting the three columns carefully and pattern matching, replacing all surnames with full names. It is worth pointing out that even when we annotated by hand, similar kinds of errors had to be spotted to reconcile the work of different team members.

### First iteration

The process extracted 1,861 unique statements from 129 articles. There were initially 946 unique ‘verbs’ or predicates in the statements. We created a knowledge graph embedding model by loading the knowledge graph into Ampligraph 1.4.0 and creating a model with the same parameters as in Graham *et al.* (2023). ^15^

The quality of the knowledge graph embedding model can be assessed using two statistics, the mean reciprocal ranking (MRR), and ‘hits@’. We split the statements into training and testing subsets (using 80% of the statements for training and 20% for testing). To calculate the first metric, triplets are ‘corrupted’ so that one element is removed. Then, the algorithm tries to predict, based on its training, what the missing element would be. The predicted answers are ranked, with the mean reciprocal ranking being the average of the rank of the correct answer among all possible answers. These scores are added up then divided by the total number of positive triples. The higher the score, the better the performance of the model (that true statements will be predicted). The second metric, Hits@10, @3 and @1 indicates the number of times a true triple ranked in the top 10, top 3, and top 1 results.

Initially, these results do not look promising; on the raw, uncorrected text-as-extracted by GPT, we get:

MRR: 0.01 ; i.e., 1% of the time the predicted answer was a true statement

Hits@10: 0.02 ; i.e., 2% of the time the predicted triple ranked in the top 10 results

Hits@3: 0.01

Hits@1: 0.01

### Second iteration

With our manually created dataset, the first time we generated a knowledge graph embedding model from it, we found similarly extremely low MRR and Hits scores. There was too much variability in the verbs/predicates in our triples. We found that if we rationalized the verbs such that synonyms or similar concepts were used instead of the complete original phrasing—in effect, creating a data model of the relationships expressed in the unstructured text—we ended up with a more restricted list of verbs capturing the essential relationships within this network. For our manually created dataset, we used MS Excel’s pivot table function to tally up the various predicates, enabling us to see at a glance conceptually similar statements that we could then edit or rationalize. This also allowed us to introduce some nuance into the knowledge graph. For example, consider these two triplets that use the same predicate, ‘bought from’:

        Person_a, bought from, Person_b

        Figurine, bought from, Person b:

That phrase, ‘bought from’ is doing subtly different grammatical work in the second statement. In which case, we altered it to read,

        Person b, sold, Figurine

We did the same process on the results generated by GPT-3. As part of this clarification process (the same process we followed in Graham *et al.*, 2023) we also expunged statements around cultural affinities or art historical statements, the locations of sites or cultures, and their date ranges, leaving us with a knowledge graph consisting of actors, objects, and organizations in the trade.

After this process, there were 888 statements in the graph with 66 predicates, which is on the same order as with our earlier work. We regenerated the knowledge graph embedding model, divided into training and testing sets, and calculated the statistics:

        MRR: 0.44

        Hits@10: 0.48

        Hits@3: 0.45

        Hits@1: 0.42

While these scores are much lower than what we reported for our hand-annotated and curated knowledge graph, they compare well with Ampligraph’s own demonstration dataset ^16^ culled by hand from Game of Thrones character relationships: ^17^

        MRR: 0.46

        Hits@10: 0.58

        Hits@3: 0.53

        Hits@1: 0.38

The factor here is the data model: if we spent more time clarifying and curating the predicates, we suspect we would have higher scores. On the other hand, part of the point of this experiment is to see what can be accomplished with a minimum of manual intervention. SG spent approximately one workday on clarifying the model to introduce the nuance described above. We regenerated the model and found that we were able to increase the MRR score from 0.01 to 0.44.

## Discussion

It is worth reminding the reader that this model is a refraction of reality, and a way of generating useful hypotheses or educated guesses in a faster and more systematic way than we could produce on our own. The Ampligraph package allows us to treat each entity, and each predicate, as a kind of building block that it can systematically combine together into statements and evaluate the likely probability that the statement is true. We have come to think of these as tips that the police get from a well-informed informant. The police do not know if the tips are going to pan out, but because the informant has good info to work with, the tips can be used to target investigations.

The general statistics used to evaluate the knowledge graph embedding model are similar, if weaker, to the ones generated from the embedding model of our custom hand-annotated knowledge graph. This reduction in overall quality might be acceptable if the automatically-extracted knowledge graph, transformed into an embedding model, generates similar hypotheses to our original model. That is, do we get similar results? If we do, then this process represents a serious acceleration of the workflow which opens up a wider mass of data to our analysis and makes the method more practical and implementable in a real-world setting.

To answer the question ‘Do we get similar results?’, we fitted a model using the same parameters to the entire dataset (rather than just 80% as we used for training the first time for generating the evaluation metrics in the previous section) and proceeded to interrogate it. In Table 1 below are statements that we used to test our original model from hand-annotated data, statements that were not in the original graph, but a mix of likely and unlikely hypotheses.

**Table 1.** Test statements for the knowledge graph embedding model and the resulting metrics.

Statement	Rank	Score	probability
gianfranco becchina worked with ali and hicham aboutaam	4	2.50	0.92
fritz bürki sold to leon levy	120	0.53	0.63
giacomo medici worked with marion true	220	0.48	0.62
robert hecht sold to barbara and lawrence fleischman collection	443	0.25	0.56
marion true bought from giacomo medici	450	0.01	0.50
giacomo medici sold to marion true	656	-0.18	0.46
roger cornelius russell yorke bought from robin symes	1259	-0.30	0.42

These resulting ranks, scores, and probabilities are largely similar to what our hand-curated dataset produced, with some subtle differences.

The hypothesis that antiquities trafficker Gianfranco Becchina ^18^ worked with antiquities dealers Ali ^19^ and Hicham Aboutaam ^20^ is returned by the model with a very strong probability. Remember, there is no such statement in the original encyclopedia articles from the Trafficking Culture website. Here, the model is extrapolating from the similarity of Becchina’s patterns in the knowledge graph which we know are very similar to Giacomo Medici’s because Medici *did* have dealings with the Aboutaam brothers according to the Trafficking Culture Encyclopedia. A photographic archive of Polaroid photographs taken by Becchina of artefacts that passed through his hands that was later seized by police does show some artefacts that he handled eventually being offered for sale by the Aboutaam brothers’ Phoenix Ancient Art dealership, ^21^ so this hypothesis seems plausible. It is important to remember that neither we nor our model are alleging criminal activity. Rather the model has simply suggested that it is likely that Becchina and the Aboutaam brothers, three men operating at the same time with dealings in classical antiquities and with significant ties to Switzerland are described in ways that could suggest they may have worked together at some point. ^22^

The next five hypotheses have much weaker probabilities associated with them. Nevertheless, these hypotheses mostly conform to our pre-existing knowledge about relationships in the antiquities trade. In our hand-annotated model, the probabilities were much higher (on the order of .90). In this case, our model is still serving up statements that seem plausible, but if this were the *only* model we had, we might not put too much emphasis on these statements. Finally, the least likely statement again conforms with our existing knowledge in that the model considers it unlikely. Yorke and Symes were based in different countries and dealt in different kinds of antiquities sourced from different parts of the world, and we would not expect there to be much overlap or connection between them.

Finally, we visualize the 400-dimension knowledge graph embedding model through dimensionality reduction (using the UMAP algorithm) to look for clusters of entities, focussing again on two major players in the antiquities trade who we evaluated in our original paper: antiquities dealers and convicted traffickers Giacomo Medici and Leonardo Patterson. Medici’s antiquities dealings focused on classical antiquities, while Patterson concentrated on Latin American antiquities. In our previous study, the visualization of the model suggested connections for Medici that accorded well with our specialist domain knowledge. For Patterson, it suggested both connections that we were aware of and others that were unknown to us. Subsequent research did confirm the some of the previously-unknown relationships suggested by the model, in particular, a connection between Patterson and the Brooklyn Museum.

The first figure depicts that three-dimensional space and the closest points to the ‘Leonardo Patterson’ entity, as known in the knowledge graph. The Brooklyn Museum is amongst the top six closest entities in this embedding model; this is the same connection that our hand-annotated dataset and model surfaced which led to the fruitful research strain summarised in out introduction. ^23^

**Figure 1.**
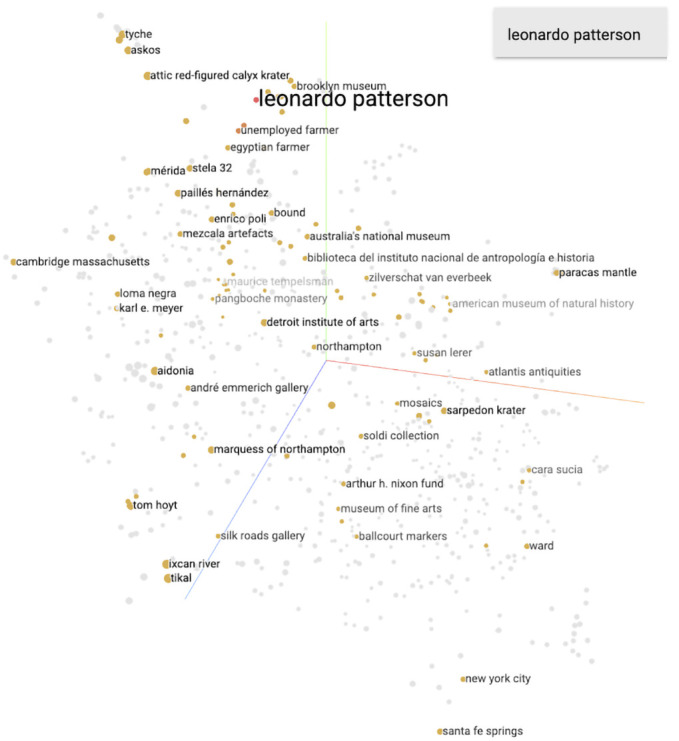
The 400-dimension knowledge graph embedding model reduced to three dimensions via the UMAP approximation, with the closest points (via cosine distance) to ‘Leonardo Patterson’ highlighted.

Closest entities in the Figure 1 visualization:

        marjorie neikrug 0.627

        glenn rittenour 0.628

        andré emmerich 0.788

        brooklyn museum 0.877

        american museum of natural history 0.923

        loma negra 0.923

        karl e. meyer 0.923

For Giacomo Medici, the closest entities mostly were connected with the Getty Museum and its officers, ^24^ as well as some of his business partners.

**Figure 2.**
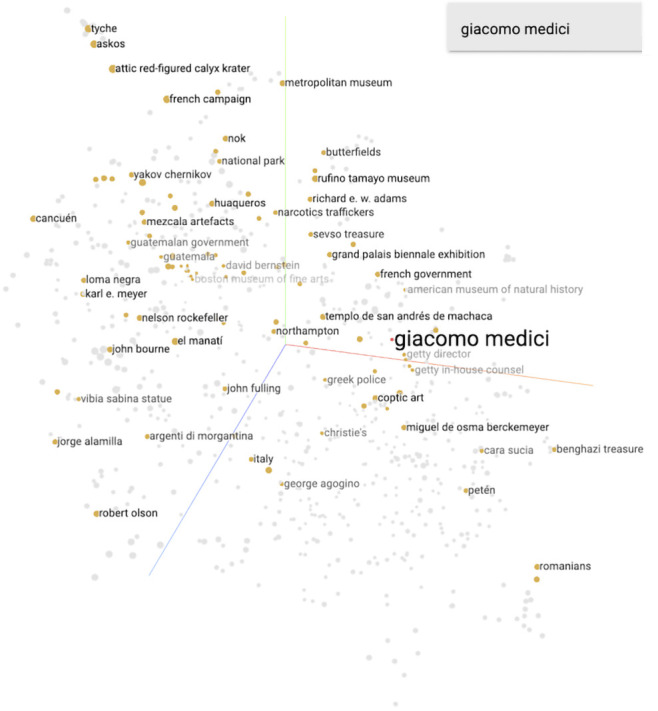
The 400-dimension knowledge graph embedding model reduced to three dimensions via the UMAP approximation, with the closest points (via cosine distance) to ‘Giacomo Medici’ highlighted.

Closest entities in the Figure 2 visualization:

        getty director 0.693

        getty board of trustees 0.700

        getty in-house counsel 0.704

        getty ceo 0.712

        david bernstein 0.761 ^25^

        george ortiz 0.790

        robert hecht 0.811

        metropolitan museum 0.895

That the automatic entity extraction via GPT-3 to knowledge graph thence to knowledge graph embedding pipeline provides very similar results as our hand-annotation model would seem to confirm for us that this workflow provides valid results, but with an enormous benefit of speed and amount of data able to be processed. Further experimentation with framing the prompt to be more efficient in terms of numbers of tokens used will reduce the financial cost of using GPT-3. Turning the creative temperature down to 0 will probably also reduce some of the sources of error. Assessing the results and correcting errors took less time with the GPT-3 results than it did with the hand-annotated results (one workday, versus several) since the nature of the errors were consistent and could be picked out quickly by eye.

Moving forward, we have modified Danny Richman’s tutorials for integrating GPT3 into a Google Spreadsheet ^26^ to use our prompt. This enables us to set up a pipeline where sources of information (newspaper articles, journal RSS feeds, basic webscraping) can be ingested semi-automatically, with the resulting knowledge graph built automatically. The knowledge of the trade that we have, as subject specialists and as captured in the Trafficking Culture and other database can be augmented with other sources of knowledge, making this combination of tools and approaches a powerful new advance for dealing with this shadowy trade.

There are of course limitations to this semi-automatic approach for generating the basic knowledge graphs, chief amongst them the difficulty of creating a prompt that generates the right kind of result, one that consistently picks out the important entities and consistently identifies the right relationships. A demonstration by developer Max Woolf for using GPT-3 to categorize the sentiment of short texts seems promising in how it constrains GPT-3 to select categories from a provided list ( Woolf, 2023); perhaps it could be adapted so that GPT-3 identifies those relationships from an investigator’s list (which would mean less curation of the results). As of this writing, the even larger and more capable GPT-4 model is being made available to early adopters. It might be that a larger model solves many of these issues simply by virtue of its size. Further research exploring the framing of prompts for particular kinds of tasks useful in this field (or in archaeology or cultural heritage more broadly) might begin to develop useful patterns that enable us to understand how, when, and why one might use large language models productively. If researchers kept a common repository of useful prompts, with their limitations and strengths, we could have a very powerful tool for dealing with masses of material at scale.

## Conclusion

While there is a reduction in the quality of the model, the result of this entity extraction workflow powered by the GPT-3 large language model ultimately produced results similar to what we achieved through a manual hand-annotation and curation process. The resulting savings of time, even when error correction and rationalization is factored in, is orders of magnitude faster. The trade-offs therefore seem worth it, as long as we think of the results of the model as ‘tips’ or ‘hypotheses’ for further research conducted by domain experts. The model does not produce statements of ‘truth’ so much as ‘hey, did you ever think to look at…’

Moving forward, we will continue the alchemical experiments to find prompt language (so called ‘prompt engineering’) that can produce better results with fewer errors. The ability to tell the machine what we are after, in plain language, and have workable and meaningful results a few minutes later will be a game changer for the kind of work we and others in the fields of illicit antiquities research and art market studies are doing. The clues strewn through newspaper articles, auction catalogues, court documents, transcribed interviews, provenance records, and other kinds of unstructured data sources (contrasting here with formal databases), coupled with knowledge graph embedding models, means that we will have a powerful tool for surfacing potential connections and other leads in the illegal and illicit antiquities trade as well as other less sinister domains. One of the grand challenges for archaeology’s grand challenges—automatic knowledge extraction—now seems within reach.

## Data Availability

No data associated with this article.

## Footnotes

^1^ A useful discussion of this technology is at Muller, 2022

^2^ DY is a member of Trafficking Culture and wrote many of the original articles

^3^ Different models use different encoding processes.

^4^ Weng, 2023 has an excellent summary and examples of various approaches to devising prompts: https://lilianweng.github.io/posts/2023-03-15-prompt-engineering/).

^5^ Those within the Trafficking Culture Encyclopedia, available at: https://traffickingculture.org/encyclopedia/

^6^ Two key figures in a well-studied antiquities trafficking network, see https://traffickingculture.org/encyclopedia/case-studies/giacomo-medici/

^7^ From https://traffickingculture.org/encyclopedia/case-studies/giacomo-medici/

^8^ Although we wonder whether the working conditions of Mechanical Turkers might be a complicating factor; this was not considered in the analysis.

^9^ And the pricing for using GPT-3 depends on the number of tokens

^10^ The next iteration model, GPT-4, which accepts larger texts, is not publicly available at the time of writing.

^11^ A solution to this problem could be to employ a natural language processing technique called ‘coreference resolution’, which seeks to replace pronouns or other indefinite words with the entities that the words refer to. However, this is not an easy problem to solve and is a current focus of work for us; the trick is to validate the text to make sure that the *right* pronouns have been correctly replaced.

^12^ The use of graph databases to manage and query the knowledge graph is a dimension to this experiment that will be explored and explained in a follow-up paper.

^13^ https://github.com/varunshenoy/GraphGPT

^14^ Other projects in development such as LlamaIndex use clever integrations to enhance prompting such that large volumes of data can be indexed and processed into knowledge graph triplets at once, see https://gpt-index.readthedocs.io. We did not become aware of LlamaIndex until comparatively recently, but it looks like a promising approach.

^15^ An important caveat here is that when we began our experiment the official Ampligraph package only worked with the now deprecated Tensorflow 1 libraries. An unofficial port of Ampligraph configured to work with Tensorflow 2 is available from Github.com user ‘arylwen’ at https://github.com/arylwen/AmpliGraph. We tested this version of Ampligraph on our original work and it returned the same results as before, so we assume that no errors have been introduced into the package via this port. As we finished writing this paper, the official Tensorflow 2 version, ‘Ampligraph 2’, was released, which has a different data input/output pipeline (see https://github.com/Accenture/AmpliGraph). We did not use Ampligraph 2.

^16^ https://docs.ampligraph.org/en/1.4.0/tutorials/AmpliGraphBasicsTutorial.html

^17^ https://github.com/neo4j-examples/game-of-thrones

^18^ https://traffickingculture.org/encyclopedia/case-studies/gianfranco-becchina/

^19^ https://www.theartnewspaper.com/2023/01/27/antiquities-dealer-ali-aboutaam-given-18-month-suspended-sentence-by-geneva-court

^20^ https://www.theartnewspaper.com/2004/09/01/lebanese-antiquities-dealers-prosecuted-in-us-and-egypt

^21^ e.g., as presented on a page on the Trafficking Culture website that was not analysed: https://traffickingculture.org/data/tracking-illicit-antiquities/four-antiquities-offered-at-christies-london-oct-1-2015-which-appear-to-be-in-photo-archived-seized-in-police-raids/

^22^ Observers of the antiquities trade are likely to read this statement and say “well, obviously.” While the possibility of a working connection between Becchina and the Aboutaam brothers is unlikely to surprise anyone, the model generated this suggestion despite the fact that no such connection exists in the Trafficking Culture Encyclopedia. In this instance “obviously” is good as it provides a check on our model’s ability to produce good tips.

^23^ As an interesting aside, the present GPT-3 powered approach visualized here also suggests a link between Patterson and the American Museum of Natural History which hitherto we had no reason to suspect. Preliminary investigation does demonstrate object donations by Patterson to this institution as well, which we will discuss in a follow-up paper.

^24^ This is a well-known connection, see Felch and Frammolino (2011) and Watson and Todeschini (2007).

^25^ The reader should note that we consider this to be an unlikely connection. Bernstein is a dealer in Latin American ancient objects who pleaded guilty to charges of filing a false customs declaration related to Peruvian objects in 1982 (see https://traffickingculture.org/encyclopedia/case-studies/peruvian-antiquities-seized-at-dulles-airport-1981/). In 1994 he attempted to sell jewellery looted from the Peruvian site of Sipán at Sotheby's ( https://traffickingculture.org/encyclopedia/case-studies/sipan-ornaments-offered-for-sale-at-sothebys-in-1994/). While this is during the time that Medici operated, we do not believe these worlds substantially overlap.

^26^ https://www.seotraininglondon.org/gpt3-google-sheets-free-tutorial/

## Software availability

Source code available from: https://github.com/XLabCU/gpt3-relationship-extraction-to-kg

Archived source code at time of publication: https://doi.org/10.5281/zenodo.7860733. ( Graham, 2023).

License: The original code for ‘python_api.py’ is copyright Sixing Huang released under the MIT License. Our additional code (‘split_resize.sh’) is released under the MIT No Attribution License.
