# Supplementary material for: Investigating antiquities trafficking with generative pre-trained transformer (GPT)-3 enabled knowledge graphs: A case study
Source: Open Res Eur. 2023 Jun 20;3:100. [Version 1] doi: 10.12688/openreseurope.16003.1 (PMC10445804; doi:10.12688/openreseurope.16003.1)
Supplement: Peer review history

# Peer review: Reviewer response for version 1

**DOI:** 10.21956/openreseurope.17280.r32965

**Published:** 2023-07-20

**Authors:** Martin Berger, Alex Brandsen

**Affiliations:** 1 National Museum of World Cultures, Leiden University, Leiden, South Holland, The Netherlands; 2 Archaeology, Universiteit Leiden, Leiden, South Holland, The Netherlands

This is a great article, very clear and to the point, and we very much enjoyed reading it. The paper discusses the use of the language model GPT-3 to create a knowledge graph related to the antiquities trade. By providing GPT-3 with a prompt, the model is guided to identify knowledge statements from various sources, such as newspaper articles and auction catalogues, and create summaries in the form of subject-predicate-object relationships. The automatic process of generating the knowledge graph using GPT-3 yields comparable results to the manual approach but saves significant time and allows consideration of a larger volume of materials. This approach demonstrates the potential of using computational means to work with archaeological knowledge from diverse sources like grey literature, reports, and articles. We look forward to seeing these methods applied to other and/or larger datasets, as we think this could lead to interesting new insights.

 While we recommend for this paper to be Approved, we do have a couple of minor points that could be clarified to further strengthen the paper:

 In the baseline section, it is mentioned that “In a conventional automated approach, one might use an existing dictionary (or construct one from scratch) of named entities that would then be used to parse the unstructured text. However, if one does not know all of the relevant entities beforehand, [...] this approach might miss or improperly categorize important data.”, it is also mentioned further down that “NER [...] depends on us knowing possible named entities in advance”. Unless you mean entity types, which do need to be defined in advance, all machine learning based NER methods are capable of finding entities that are not in a dictionary or the training data. It would be good to clarify this in the text.

 This is more of a suggestion for future work than a comment, but in the section “Preprocessing the text” you mention that bigger files are split up and that relationships that span the break might be missed. Another way of possibly dealing with this would be to make the breaks overlap (by e.g. a paragraph or 2), and remove the duplicate relations afterwards.

 In the second iteration, you manually rationalised the verbs using Excel, and this took about a day for this dataset. Do you think this is scaleable to (much) larger datasets, in terms of time needed, complexity, and the ability of Excel to handle big datasets (>10k rows)?

 From the text it is not clear to us what happens to entities / nodes that have the same string/name but could be different objects. For example, you mention “Person b, sold, Figurine”, does the “Figurine” node get linked to all nodes that are called ‘figurine’, or are they all seen as separate? For e.g. names and organisations this is much clearer, and all “FBI” nodes can be seen as the same entity, but with object classes it is much more difficult to determine whether 2 similar strings are talking about the same, or separate objects.

 Another suggestion would be to reconsider the dimensionality reduction of figures 1 and 2, which have now been reduced to three dimensions, but can not be interactively examined (only seen in 2D-printed version). For a layperson, it is quite hard to understand how e.g. Leondardo Patterson can be closer to Karl E. Meyer than to the Brooklyn Museum, even though, in the visualization figure, Patterson seems to sit much closer to the Brooklyn Museum than to Meyer/Loma Negra. Maybe a reduction to two, rather than three, dimensions would be helpful for the non-expert reader to understand how these entities relate to each other?

 A final comment regards the use of possibly sensitive (both ethically and privacy/GDPR-related) data in open-source LLMs. You mention that you have used the proprietary version of the GPT, but that “open source LLMs are now being developed, and so the general approach we use here should translate to those LLMs in due course.” One can wonder whether inputting sensitive data into free, corporate-owned LLMs (which might reuse these data as training material) can raise questions regarding ethics/privacy. While it is true that ChatGPT has recently introduced the option of ‘not saving sessions’ (so that material will not be used for training), it is not clear how this will develop in the future, also among OpenAI’s competitors. 

 In all, we would like to stress again that the comments above are mere suggestions for further reflection and in no way obstacles to accepting the article, as we think the research is highly valuable and promising, and that the article is written in an accessible and clear style.

If applicable, is the statistical analysis and its interpretation appropriate?

Not applicable

Is the study design appropriate and is the work technically sound?

Yes

Is the work clearly and accurately presented and does it engage with the current literature?

Yes

Are the conclusions drawn adequately supported by the results?

Yes

Are sufficient details of methods and analysis provided to allow replication by others?

Yes

Are all the source data and materials underlying the results available?

Partly

Reviewer Expertise:

Natural Language Processing, Archaeology, Art Market Studies, Provenance Research

We confirm that we have read this submission and believe that we have an appropriate level of expertise to confirm that it is of an acceptable scientific standard.

# Peer review: Reviewer response for version 1

**DOI:** 10.21956/openreseurope.17280.r32966

**Published:** 2023-07-20

**Authors:** Dante Abate

**Affiliations:** 1 Department of Resilient Society, ERATOSTHENES Centre of Excellence, Limassol, Limassol, Cyprus

In this study, GPT-3 is used to highlight hidden links related to the illicit traffic of cultural property automatically analyzing a corpus of articles available on the Trafficking Culture website.

 The model appends text to each article, summarizing the knowledge in the form of subject, predicate, and object relationships, creating a knowledge graph. This automatic process is compared with a manually annotated knowledge graph. The knowledge graphs are then projected into a multi-dimensional embedding model using a neural network, allowing the positioning of entities to indicate the probability and type of connections. By interrogating the embedding model, new relationships and insights about the antiquity trade can be discovered, suggesting potential research avenues.

 The authors clearly and accurately presented their study with proper references to the updated literature.

 The study presented is well designed with an appropriate level of technical details which, alongside with the provided opensource code, can allow replication by others scientist.

 A statistical analysis and its interpretation appropriate about the accuracy of the results is provided. For a better understanding, these data (pages 9, 10, and 11) could be organised in tables.

 The results effectively support the conclusions drawn, although there remains a minor concern about the limited size of the training dataset. However, the promising outcomes obtained partially mitigate this concern. An interesting avenue for further investigation could involve generating synthetic data and applying the proposed methodology on a larger scale. It is important to note that this suggestion falls outside the scope of the article under review.

 In order to improve visual understanding, the authors may want to consider emphasizing the entries mentioned on page 11 within the GPT-3-driven graph. This visual emphasis would assist readers in easily identifying and comprehending those particular elements.

 Furthermore, if space permits, including a visual comparison between the manually constructed knowledge graph and the one generated through GPT-3 would be valuable for better understanding the similarities and differences between the two approaches.

If applicable, is the statistical analysis and its interpretation appropriate?

Yes

Is the study design appropriate and is the work technically sound?

Yes

Is the work clearly and accurately presented and does it engage with the current literature?

Yes

Are the conclusions drawn adequately supported by the results?

Yes

Are sufficient details of methods and analysis provided to allow replication by others?

Yes

Are all the source data and materials underlying the results available?

Yes

Reviewer Expertise:

Illicit Traffic of Cultural Property, Digital Technologies, Cultural Property Protection

I confirm that I have read this submission and believe that I have an appropriate level of expertise to confirm that it is of an acceptable scientific standard.
